# Heat in the southeastern United States: Characteristics, trends, and potential health impact

**DOI:** 10.1371/journal.pone.0177937

**Published:** 2017-05-16

**Authors:** Jeremy E. Diem, Christine E. Stauber, Richard Rothenberg

**Affiliations:** 1 Department of Geosciences, Georgia State University, Atlanta, Georgia, United States of America; 2 School of Public Health, Division of Environmental Health, Georgia State University, Atlanta, Georgia, United States of America; 3 School of Public Health, Division of Epidemiology and Biostatistics, Georgia State University, Atlanta, Georgia, United States of America; Universidade de Vigo, SPAIN

## Abstract

High summer temperatures in extratropical areas have an impact on the public’s health, mainly through heat stress, high air pollution concentrations, and the transmission of tropical diseases. The purpose of this study is to examine the current characteristics of heat events and future projections of summer apparent temperature (AT)–and associated health concerns–throughout the southeastern United States. Synoptic climatology was used to assess the atmospheric characteristics of extreme heat days (EHDs) from 1979–2015. Ozone concentrations also were examined during EHDs. Trends in summer-season AT over the 37-year period and correlations between AT and atmospheric circulation were determined. Mid-century estimates of summer AT were calculated using downscaled data from an ensemble of global climate models. EHDs throughout the Southeast were characterized by ridging and anticyclones over the Southeast and the presence of moist tropical air masses. Exceedingly high ozone concentrations occurred on EHDs in the Atlanta area and throughout central North Carolina. While summer ATs did not increase significantly from 1979–2015, summer ATs are projected to increase substantially by mid-century, with most the Southeast having ATs similar to that of present-day southern Florida (i.e., a tropical climate). High ozone concentrations should continue to occur during future heat events. Large urban areas are expected to be the most affected by the future warming, resulting from intensifying and expanding urban heat islands, a large increase in heat-vulnerable populations, and climate conditions that will be highly suitable for tropical-disease transmission by the *Aedes aegypti* mosquito. This nexus of vulnerability creates the potential for heat-related morbidity and mortality, as well as the appearance of disease not previously seen in the region. These effects can be attenuated by policies that reduce urban heat (e.g., cool roofs and green roofs) and that improve infrastructure (e.g. emergency services, conditioned space).

## Introduction

Extreme summer heat is a major public health concern across the Northern Hemisphere extratropics, especially in the United States. High temperatures and humidity cause heat stress by reducing the ability of the human body to transfer metabolic heat through conduction and evaporative cooling [1,2]. Heat events have a disproportionate impact on individuals 65 years of age or older, a demographic group typically lacking the physiologic capability to adequately respond to heat exposure [3]. Elevated temperature is associated with increased risk for those with pre-existing conditions and increases the risk of dying from respiratory, cerebrovascular, and some specific cardiovascular diseases, such as ischemic heart disease, congestive heart failure, and myocardial infarction [4]. Thousands of people are hospitalized for heat-related illnesses (HRIs) each year in the United States [5]; the 1995 Chicago heat wave was responsible for over 1,000 excess hospital admissions [6].

Regional acclimatization to heat contributes to reductions of heat-related mortality, with increased mortality in regions where extreme heat is less common [7]. Thus, people who reside in the northern United States may be at greater risk of dying from extreme heat [8]. While the prevalence of air conditioning may protect people from HRIs in the southern United States [9], extreme heat has been found to lead to increased mortality in the south as well [10].

Extreme heat events in the extratropics are caused by large-scale changes in the atmospheric circulation. Mid-troposphere ridging is a key atmospheric feature associated with heat waves in the extratropics: large positive 500-hPa geopotential height anomalies typically coincide with heat events in the United States [11–13]. These conditions produce subsidence, excessive solar radiation, light winds, warm-air advection, and long periods of hot conditions [14,15]. High apparent temperatures (ATs) during heat waves require moisture; for example, evapotranspiration from relatively moist agricultural fields in the central United States was a contributor to the very high ATs (e.g., maximum daily values exceeding 38°C) during the 1995 Chicago heat wave [11].

Extreme heat events tend to have larger impacts in urban areas than rural areas. Urban areas are typically warmer than surrounding rural areas, and paved surfaces and buildings are primarily responsible for the increased temperature by increasing short-wave radiation absorption, decreasing long-wave radiation loss, and increasing sensible-heat storage [16]. The intensity of these urban heat islands (UHIs) is determined by the difference in temperature between the maximum urban temperature and the background rural temperature, and UHI intensity is greatest under anticyclonic conditions during the summer [16,17]. Therefore, UHI intensity increases during heat waves [18]. Urban poverty, especially in the 65+ age group, has been shown to be the most influential variable on spatial variations in mortality resulting from heat waves [19]. Moreover, urban residents report a reluctance to leave windows open due to neighborhood crime or violence [3].

High ground-level ozone concentrations often occur during heat waves in the United States. Throughout much of the eastern United States, ozone concentrations during heat waves are at least 20% higher than the summer average [20]. High ozone concentrations irritate the lungs and thus affect respiratory function, especially among people with asthma [21]. Migrating anticyclones in the summer, which typically produce clear skies, light winds, and high temperatures, are associated with high ozone concentrations [22–24]. Anticyclonic conditions are favorable to the photochemical production of ozone [25]; for example, increasing temperatures increase ozone-precursor emissions and alter chemical reactions thereby resulting in increased ground-level ozone concentrations [26]. Since ozone is affected by temperature, it has been labeled a causal intermediate in the heat-mortality association [27].

High summer temperatures are also favorable for mosquitos capable of transmitting arboviruses. Within the continental United States, *Aedes (Stegomyia) aegypti* (L.), the primary urban vector of dengue and yellow fever viruses [28], is currently confined to the southern United States extending up the Atlantic coast to New York [29]. Temperature has a major effect on the capacity of a population of mosquitoes to transmit viruses: temperature is positively correlated virus transmission (i.e., increasing temperatures increase the number of blood meals in a typical mosquito’s remaining lifespan) and negatively correlated with the time required for development of virus inside a mosquito [30]. In addition, the summer season needs sufficient rainfall and relative humidity so that water levels remain adequate in outside containers (e.g., discarded tires) for egg-laying and development of immatures [28].

Climate change has the potential to increase heat-related issues throughout the United States, especially in the southeastern United States. The Southeast, which we define in this paper as Alabama, Florida, Georgia, Mississippi, North Carolina, South Carolina, and Tennessee, is already a rapidly growing region: it currently has a population of roughly 57 million, and it has grown by 61% since 1980, whereas the rest of the United States only grew by 36% [31]. With summer temperatures expected to significantly increase globally over the rest of the century [32] and a relative paucity of information about contemporary atmospheric characteristics of heat waves in the Southeast, especially in the context of apparent temperature (AT), the purpose of this study is to examine the current atmospheric characteristics of extreme heat days (EHDs) and develop future projections of summer AT–and associated health concerns–in the southeastern United States. The main objectives of the study are as follows: (1) examine the spatial and interannual variability in summer AT; (2) determine the synoptic and air-quality characteristics of extreme heat days (EHDs); (3) develop mid-century projections of summer AT under multiple scenarios; and (4) address the potential for public health impact and identify potential prevention goals.

## Materials and methods

**Fig 1:**
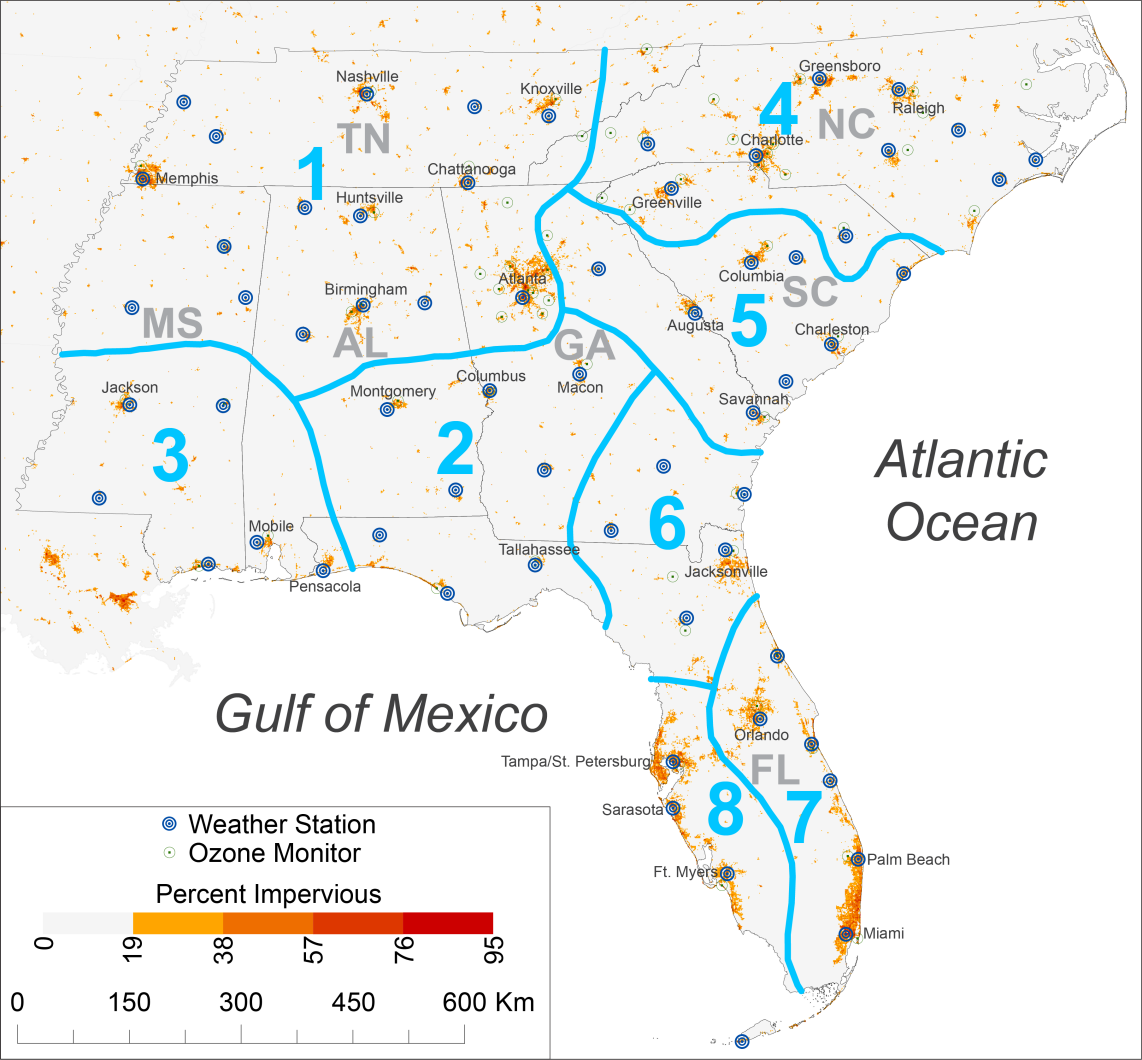
The southeastern United States and the apparent-temperature regions within it. The 63 weather stations and 63 ozone monitors for which data were obtained are shown in addition to the urbanization of 1-km^2^ cells based on percent impervious surface. Impervious-surface data, originally at 30-m resolution, were obtained from the Multi-Resolution Land Characteristics consortium.

**Fig 2:**
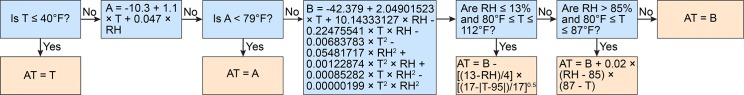
Flowchart for calculating apparent temperature (in °F). Temperature (T) is in degrees Fahrenheit and relative humidity (RH) is in percent. The flowchart is based on previous representations of the equations used to calculate the heat index [33,35].

Mean daily ATs were calculated for the summer season (June-August) from 1979–2015. Daily temperature and dew-point temperatures were acquired from 71 first-order weather stations from the Global Summary of the Day Dataset of the National Oceanic and Atmospheric Administration (NOAA) (Fig 1). Eight stations outside the Southeast were used so that AT surfaces created by simple kriging did not have edge effects. The National Weather Service algorithm for calculating AT [33] was used, since that algorithm has been found to be the most accurate procedure for reproducing the original tabular results [34,35] (Fig 2).

The southeastern United States was divided into homogeneous AT regions to simplify the geographical analyses. Using an S-mode input matrix consisting of 3,404 rows (one row for each mean daily AT value for June-August from 1979–2015) and 63 columns (i.e., weather stations), standardized principal components analysis was used to determine the homogeneous summer AT regions. The number of regions was determined from a scree plot of log-transformed eigenvalues. The loadings matrix was orthogonally rotated using the VARIMAX technique, and the maximum loading rule [36] was used to assign each weather station to a component (i.e., region) on which it had the highest loading.

Circulation, air-mass, and air-quality characteristics of EHDs were determined for each region. For this study, EHDs had daily regional-mean ATs above the 95^th^ percentile AT for the 3,404 days. Therefore, each region had 170 EHDs. Using North American Regional Reanalysis (NARR) data [37] obtained from NOAA, circulation patterns at 500 hPa and 850 hPa were constructed for composites of EHDs, and those patterns were compared with the mean circulation patterns for all days. Three-day back trajectories were produced for 30 randomly selected EHDs and 30 randomly selected non-EHDs using the Hybrid Single-Particle Lagrangian Integrated Trajectory (HYSPLIT) model [38]. The model was run with NARR data. The Air Resources Laboratory of NOAA provided the model and requisite data. Vertical motion was described by modeled vertical velocity. The starting heights of the trajectories were 300 m above ground level, and the starting locations of the trajectories were the centroids of the regions. Daily synoptic weather types (i.e., air masses) were acquired for stations near the centroids of the AT regions from Scott Sheridan of Kent State University (http://sheridan.geog.kent.edu/ssc.html) [39]. Chi-square tests (α = 0.01) were conducted for the frequencies of different types of trajectories as well as frequencies of moist-tropical and dry-tropical air masses. Hourly ozone concentrations from 2000–2015 at 71 continuous monitors were acquired from the U.S. Environmental Protection Agency, and mean daily maximum 8-hour average ozone concentrations were calculated at each monitor for all days and for EHDs at nearby weather stations. These particular EHDs were July-August days from 2000–2015 with ATs above the 95^th^ percentile. As was the case with the weather stations, eight stations outside the Southeast were used so that AT surfaces created by inverse-distance weighting did not have edge effects.

Statistical tests were used to examine changes in summer middle-troposphere circulation and relationships between the circulation and regional AT. The 500-hPa geopotential heights were extracted from the National Centers for Environmental Prediction–U.S. Department of Energy Atmospheric Model Intercomparison Project phase II Reanalysis-2 dataset [40]. Trends in summer 500-hPa heights over 1979–2015 were assessed using one-tailed Kendall-Tau correlation tests (α = 0.01; one-tailed). Pearson product moment correlations between regional AT and 500-hPa and 850-hPa fields were calculated.

Both the mean AT and the frequency of EHDs for each region were used to examine the interannual variability in regional ATs over the 37 years. Pearson product moment correlations between seasonal AT and EHD frequency were calculated. Trends in regional AT for the three individual months (i.e., June, July, and August) and the entire summer season over 1979–2015 were assessed using one-tailed Kendall-Tau correlation tests (α = 0.05; one-tailed). The Kendall–Theil robust line, the median of the slopes between all combinations of two points in the data [41], was used to estimate changes over the 37-year period.

Mid-century (i.e., 2040–2060) projections of summer AT were calculated from downscaled temperature and relative humidity data derived from global climate model (GCM) output. Bias-Correction Spatial Disaggregation (BCSD) monthly mean near-surface temperatures at 0.125°resolution were obtained from CMIP5 (Coupled Model Intercomparison Project 5) multi-model ensemble experiments for 1979–2060 [42]. These projections were obtained for all 71 weather stations, and each station had a total of 231 projections from the following: three representative concentration pathway (RCP) scenarios (RCP2.6, RCP4.5, and RCP8.5), up to 36 models, and up to ten runs per model. All projections were obtained from "Downscaled CMIP3 and CMIP5 Climate and Hydrology Projections" archive at http://gdo-dcp.ucllnl.org/downscaled_cmip_projections/. Relative-humidity projections at each station were derived from the Multivariate Adaptive Constructed Analogs (MACA) method [43] and obtained from the University of Idaho. The MACA method downscales model output from 20 GCMs of CMIP5; projections of relative humidity were available for the RCP4.5 and RCP8.5 scenarios. The projected RH RCP 2.6 scenario was assumed to be the mean of the current RH and the projected RH under the RCP 4.5 scenario. The difference between 2040–2060 projections and projected values for 1979–2015 were added to the observed values for 1979–2015 to obtain the final projections for 2040–2060 for the 71 weather stations.

## Results

### Spatial variations in summer AT

The principal components analysis produced eight AT regions (Fig 1, Table 1). The northernmost regions (1, 4, and 5) had the smallest mean seasonal ATs and the largest deviations between the mean AT on EHDs and the mean AT for all days. These regions also had a small percentage of EHDs occurring in June and a majority of EHDs occurring in July. Regions 2, 3 and 6, the three regions extending from southern Mississippi to northern Florida, had moderate mean ATs and deviations as well as July being the dominant month for EHDs. The two peninsular Florida regions (7 and 8) were considerably different from the other regions: they had the highest mean ATs and the smallest deviations along with the lowest percentages of EHDs occurring in July. Finally, there was relatively little difference in EHDs among the regions; the values ranged from 32.5°C for Region 4 to 35.5°C for Region 8.

**Table 1:** Apparent temperature (AT) characteristics for the eight regions.

Region	Summer AT (°C)	EHD AT (°C)	Deviation (°C)	June EHDs (%)	July EHDs (%)	August EHDs (%)
1	27.1	33.2	6.1	5	56	39
2	28.7	34.3	5.6	17	49	34
3	29.0	34.6	5.6	14	48	38
4	26.2	32.5	6.3	12	52	36
5	28.1	34.2	6.2	12	54	34
6	28.8	34.4	5.6	17	58	29
7	30.0	34.1	4.1	12	39	48
8	31.4	35.5	4.1	24	38	39

Shown are the mean summer AT, the mean AT on extreme heat days (EHDs), the deviation between the two means, and the percentages of EHDs occurring in June, July, and August.

**Fig 3:**
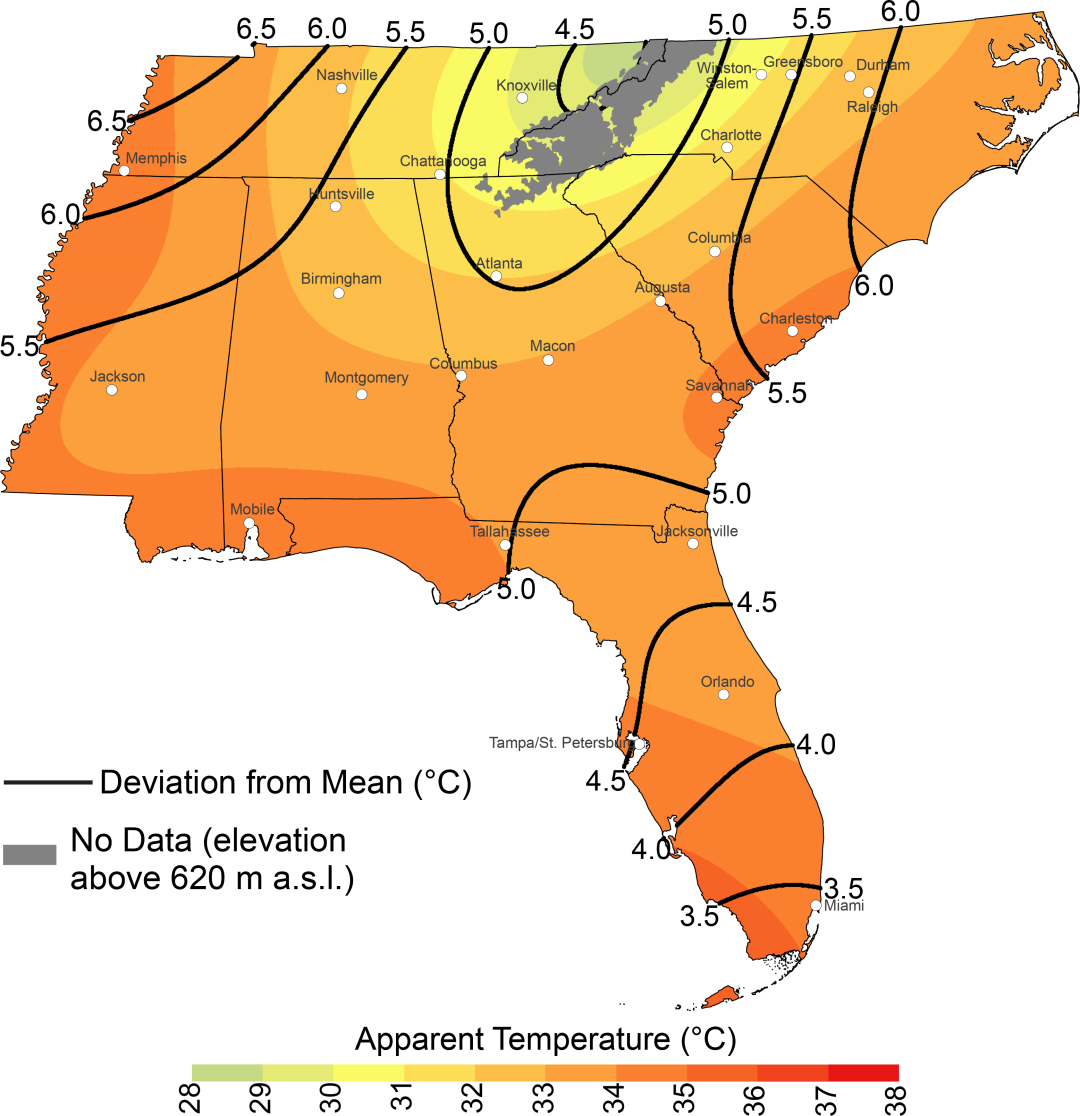
Mean apparent temperatures for extreme heat days. Values are °C and the isotherms are deviations (°C) from the mean seasonal apparent temperature.

A detailed look at EHD ATs shows decreases in AT with an increase in latitude and elevation (Fig 3). Northeastern Tennessee had the lowest values (i.e., less than 29°C), while southwestern Florida had values exceeding 35°C. Deviations from the mean summer AT generally were maximized in the northwestern and northeastern parts of the Southeast; therefore, among all the major cities, Memphis and Raleigh had the most intense EHDs (i.e., EHDs with ATs at least 6°C higher than the mean AT) when compared to mean conditions.

### Atmospheric and air-quality characteristics of EHDs

**Fig 4:**
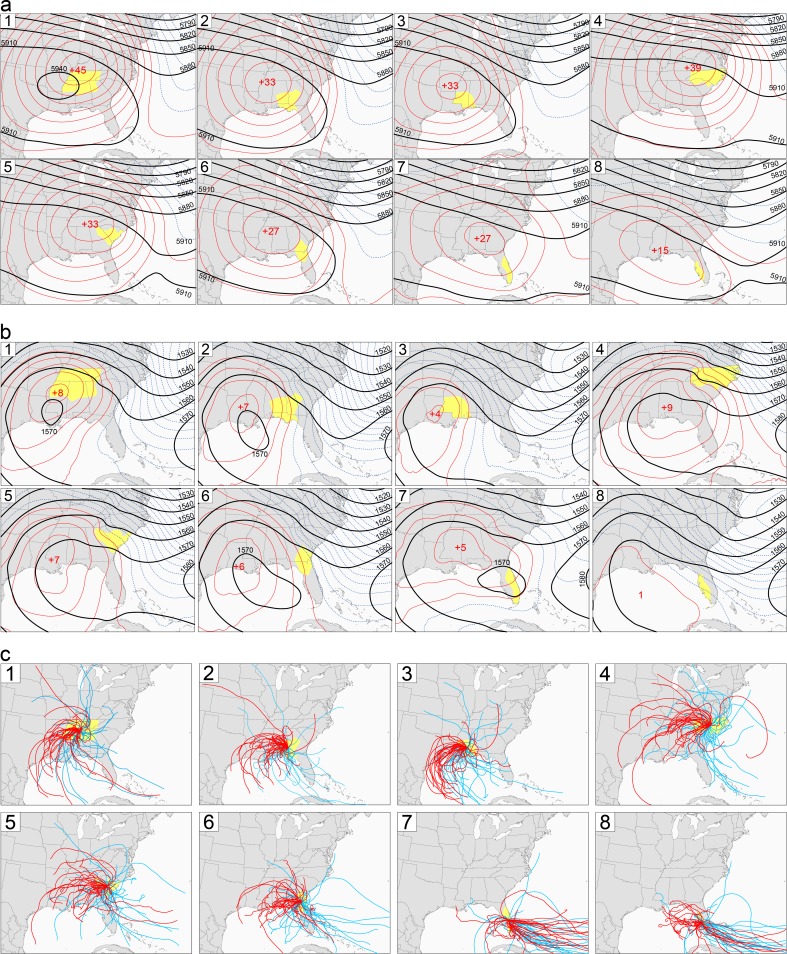
Circulation characteristics of extreme heat days. (a) Mean 500-hPa geopotential heights (black lines) on July-August extreme heat days (EHDs) for the eight regions are shown as solid black lines. (b) Mean 850-hPa geopotential heights (black lines) on July-August EHDs for the eight regions are shown as solid black lines. Positive and negative deviations from mean July-August heights are shown as solid red lines and dashed blue lines, respectively. The red numbers are the maximum positive deviations. (c) Three-day air-mass back trajectories for the eight regions. The red lines are trajectories for 30 random extreme heat days. The blue lines are trajectories for 30 random days that are not extreme heat days.

EHDs in July and August in all eight regions were characterized by ridging and anticyclones over the Southeast (Fig 4A and 4B). June was excluded from the EHD analyses because on average only 14% of the EHDs occurred in that month (Table 1). The northernmost regions had the strongest height anomalies and the southernmost regions had the weakest height anomalies. The maximum positive height anomalies for most of the regions were located either directly over or to the immediate west of the region. The middle-troposphere ridging on EHDs occurred over most of the central and eastern/southeastern United States. In the lower troposphere, EHDs were characterized by either a migratory anticyclone over the Southeast or a westward extension of the North Atlantic Subtropical High. The 850-hPa pattern is similar to the composite for dry summer days in Southeast [44]. The upstream ridging provides subsidence and associated adiabatic warming of the air [15].

With the exception of southeastern Florida, seven regions tended to have air parcels coming from the southwest to west prior to EHDs (Fig 4C). Over the 72 hours preceding an EHD, the air parcels descended on average by approximately 700 m, with the descents ranging from 340 m for Region 8 to 940 m for Region 4. Therefore, the parcels remained in the lower troposphere over the time period. Similar trajectories were found for the deadly August 2003 heat wave in Europe [45]. EHDs in Regions 1, 2, 3, and 6 had disproportionately high frequencies of trajectories extending to the southwest; these trajectories often extended back over Louisiana, Texas, and the Gulf of Mexico. The Gulf of Mexico is a major source of moisture for the Southeast [46]. EHDs in Regions 4 and 5 had a disproportionately high percentage of trajectories extending to the west with a small percentage of trajectories reaching the Gulf of Mexico; the trajectories tended to travel over more land than did EHD trajectories for the other regions. EHDs in Region 7 were unique, for the air primarily came eastward from the Atlantic Ocean. On the other hand, trajectories for Region 8 tended to originate over the Gulf of Mexico.

EHDs in all regions were characterized the presence of tropical air masses, particularly moist air masses (Table 2). All regions except for Region 5 had significantly high frequencies of moist-tropical air masses on EHDs. Region 1 was the most affected region: moist-tropical air masses were twice as frequent on EHDs compared to the rest of the days. Region 1 also had a significantly low frequency of dry-tropical air masses occurring on EHDs. This is in direct contrast to Regions 2, 4, 5, and 6, which had significantly high frequencies of dry-tropical air masses occurring on EHDs. Dry-topical air masses were the most frequent on Region 4 and 5 EHDs. The aforementioned prevalence of back-trajectories on EHDs that did not extend to the Gulf of Mexico for these two regions explains the relative lack of moist-tropical air masses and relative abundance of dry-tropical air masses.

**Table 2:** Air-mass characteristics at representative stations in the eight regions.

Region	Station	EHDs MT (%)	Rest MT (%)	EHDs DT (%)	Rest DT (%)
1	Huntsville, AL	94*	42	0*	8
2	Montgomery, AL	71*	52	13*	5
3	Jackson, MS	87*	51	10	8
4	Raleigh, NC	58*	43	37*	9
5	Augusta, GA	45	52	42*	7
6	Jacksonville, FL	85*	64	10*	3
7	Orlando, FL	94*	70	0	0
8	Tampa, FL	95*	69	0	0

Shown are the percentage of extreme-heat days (EHDs) and the rest of the days (Rest) in July-August from 1979–2015 that had moist tropical (MT) and dry tropical (DT) air masses.

Asterisks denote significant (α = 0.01) differences using Chi-Square tests.

**Fig 5:**
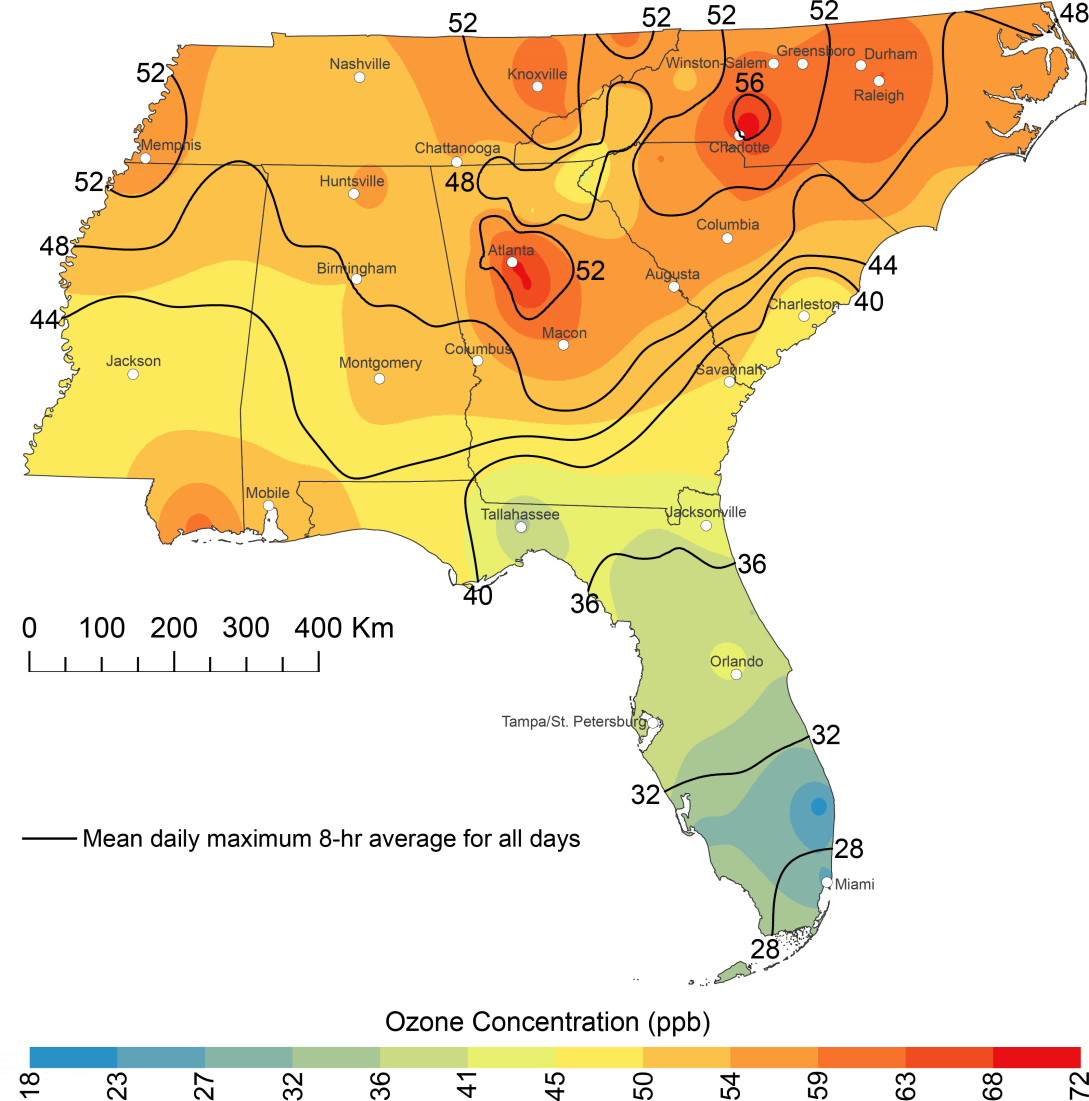
Mean daily maximum 8-hr average ozone concentrations on extreme heat days in July-August from 2000–2015. The black lines are mean daily maximum 8-hr average ozone concentrations on all days in July-August.

Ground-level ozone concentrations throughout most of the Southeast are higher on EHDs than on mean July-August days (Fig 5). The highest daily maximum 8-hr average concentrations in the Southeast on EHDs occurred over and downwind of downtown Atlanta and Charlotte and extended over other heavily populated areas (e.g., Raleigh) in central North Carolina. The concentrations over and downwind of Atlanta and Charlotte were roughly 15 ppb (30%) higher than the mean concentrations in July-August. Florida is relatively well-ventilated and mostly lacks large upwind emissions sources [47]; therefore, high ozone concentrations did not occur in Florida and multiple stations even had lower concentrations on EHDs compared to the mean.

### Interannual variability in summer AT

**Fig 6:**
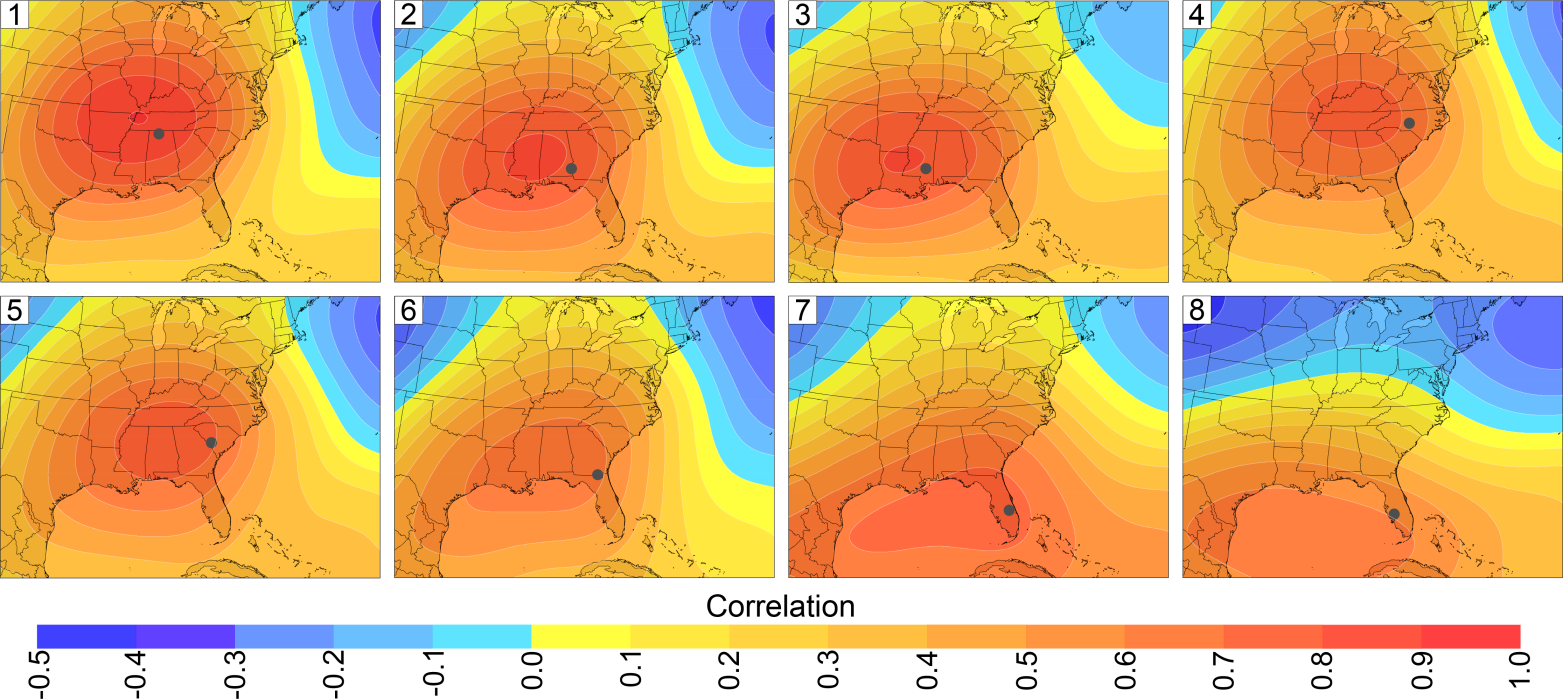
Correlation between June-August apparent temperature and 500-hPa heights from 1979–2015. The grey circles are the centroids of the eight regions.

Interannual summer AT for a region is strongly positively correlated with 500-hPa geopotential heights over and west of the region (Fig 6). The maximum correlations, which ranged from 0.68 for Region 6 to 0.90 for Region 1, were always several hundred kilometers west of the region centroids. Summers with high AT were linked to mid-troposphere ridging over the eastern and central United States. The northern and western regions (i.e., Regions 1 to 5) had the largest positive correlations with 500-hPa heights, while the southeastern regions (i.e., regions 6 to 8) had the weakest correlations. Positive correlations with 850-hPa heights were much weaker than the 500-hPa correlations (figure not shown): the maximum correlations ranged from 0.09 for Region 7 to 0.52 for Region 1.

**Fig 7:**
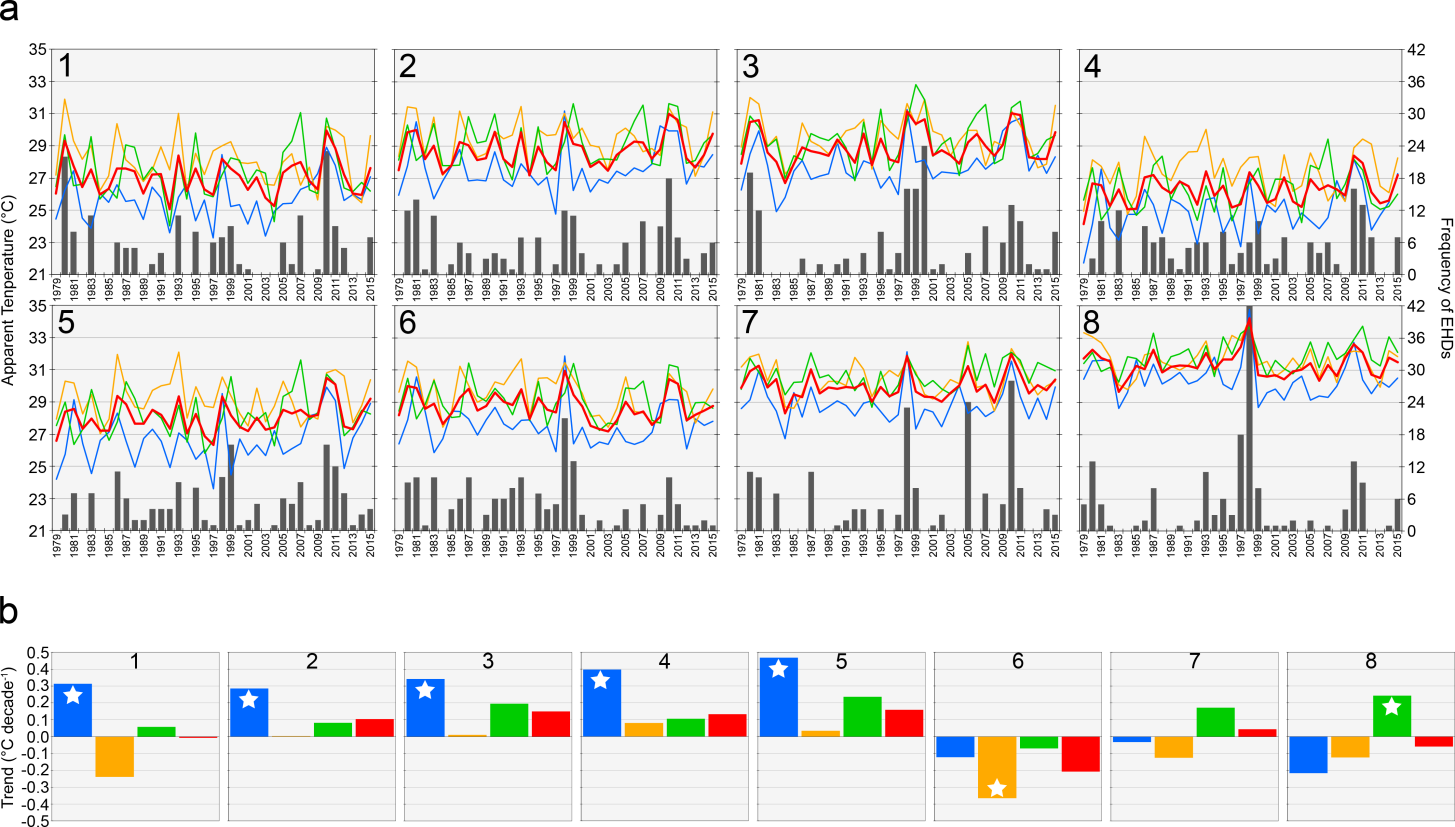
Time series and trends for apparent temperatures for the eight regions. (a) Interannual variability in June (blue), July (orange), August (green), and June-August (red) apparent temperatures and frequency of extreme heat days. (b) Trends (°C decade^-1^) in apparent temperature for June (blue), July (orange), August (green), and June-August (red). Stars denote significant (α = 0.01; one-tailed) trends.

ATs increased only modestly from 1979 to 2015 (Fig 7). There were significantly increasing June ATs in Regions 1 to 5 (i.e., the northern and western regions); the mean AT trend for those regions was 0.36°C decade^-1^. Region 6 deviated from the rest of the regions; it had significantly decreasing July and seasonal ATs (Fig 7B). The range in seasonal trends among the eight regions was -0.21°C decade^-1^ to 0.16°C decade^-1^, and the mean trend was 0.04°C decade^-1^. None of the positive trends were statistically significant.

For each region, the frequency of EHDs was highly correlated with the mean AT and the regions shared several extreme years for both. Pearson product-moment correlations between AT and EHDs ranged from 0.75 for Region 4 to 0.86 for Region 1. For the Southeast as a whole, the two most extreme years in terms of AT and EHDs were 1998 and 2010; over 20% of the EHDs over the 37-year period occurred in those two years. And in Region 8, one-quarter of all EHDs occurred in 1998. Other common extreme years across the regions were 1980, 1993, and 2011.

### Future projections of summer AT

**Fig 8:**
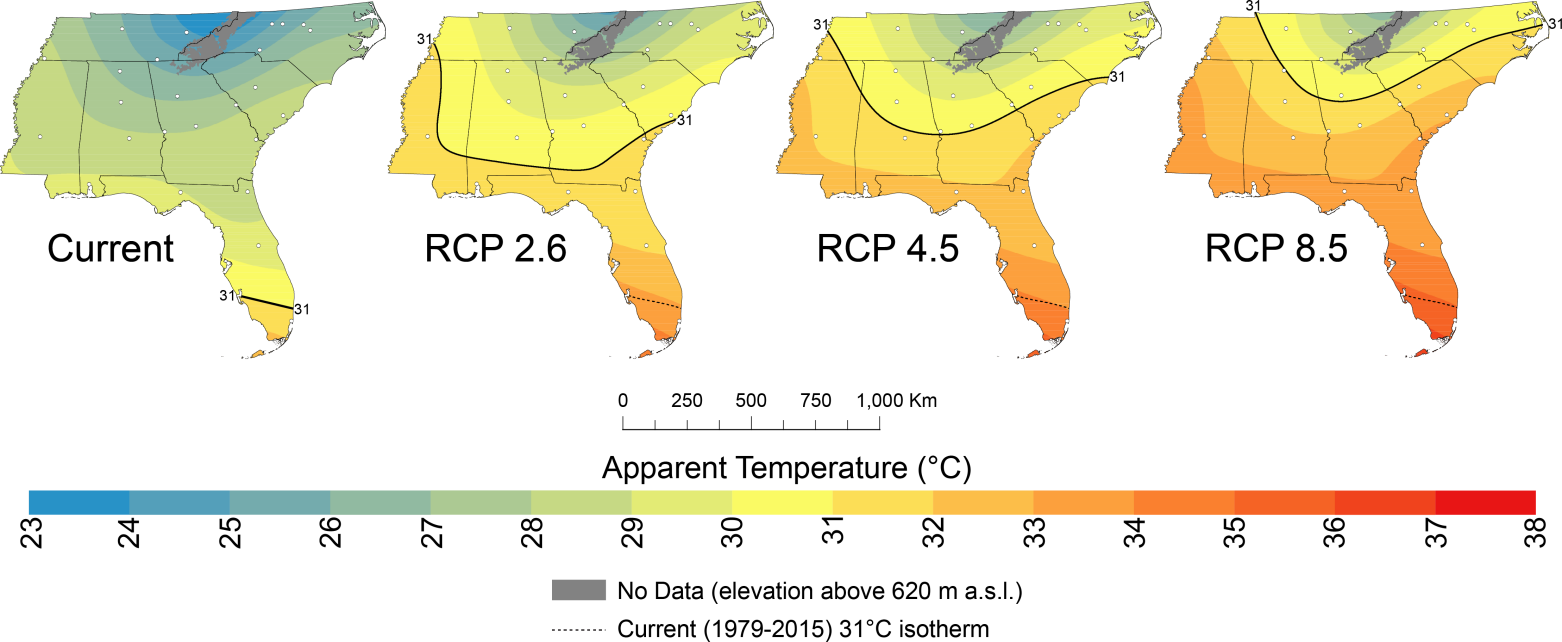
Projections of summer apparent temperatures in 2050. Projections under the RCP2.6, RCP4.5, and RCP8.5 scenarios are derived from temperature and relative humidity projections from model output using those scenarios. The solid black (dashed black) line is the projected (present-day) 31°C isotherm.

Summer ATs across the Southeast are projected to increase substantially over the next several decades, resulting in most of the Southeast having ATs similar to that of present-day southern Florida, which has a tropical climate (Fig 8). Under all scenarios, increases in ATs were projected to be uniform across the Southeast; AT increases under the RCP2.6, RCP4.5, and RCP8.5 scenarios are 2.4°C, 3.1°C, and 4.1°C, respectively. Therefore, the current AT isotherms will migrate northwards. The 31°C line, for example, which is the present-day demarcation of southern Florida, will migrate northwards by 600 km, 700 km, and 800 km under the RCP2.6, RCP4.5, and RCP8.5 scenarios, respectively.

## Discussion

Despite the large difference between northern regions and southern regions in the Southeast with respect to EHD ATs compared to mean conditions, the temperature-mortality relationship is not well-structured spatially throughout the Southeast and varies considerably among studies. Incidences of heat-related illnesses are strongly affected by the difference between temperature extremes and the mean temperature [48]. Therefore, heat-vulnerable populations in western Tennessee and northern North Carolina might be much more prone to HRIs than are other heat-vulnerable populations in the Southeast. Supporting the above hypothesis is the following: the mean summertime heat-attributable mortality rate for Greensboro, North Carolina has been shown to be 1.5 times to nearly twice as high as the rates for Birmingham, AL, Atlanta, GA, Memphis, TN, and Tampa, FL, and at least four times the rates for Jacksonville, FL and Miami, FL [49]. Other research on mortality, however, has shown that Jacksonville and Miami, along with Charlotte, NC, were the only cities out of 11 cities examined in the Southeast with definitive mortality increases associated with heat events compared to non-heat event days; Miami had the strongest temperature-mortality relationship [50]. In another study that included five southeastern cities, Atlanta had a stronger temperature-mortality relationship than Jacksonville, Tampa, and Miami, whereas a relationship did not exist for Charlotte [51]. And another study also has shown that Atlanta had much higher heat-mortality rates in recent decades than did Charlotte, Miami, and Tampa [9].

Increased ozone-precursor emissions and atmospheric transport caused extremely high ozone concentrations to occur near Atlanta and throughout much of central North Carolina on EHDs. Mean concentrations downwind of downtown Atlanta and Charlotte equaled or exceeded 70 ppb, which is the 2015 National Ambient Air Quality Standard (NAAQS) for ozone [52]. Local emissions caused the high ozone concentrations in Atlanta and Charlotte [53,54], which are the two largest urban areas outside of Florida. The predominant westerly flow on EHDs (Fig 4C) caused the highest ozone concentrations to be over and east of downtown Atlanta and Charlotte.

Extremely high seasonal AT in the Southeast might be linked to Pacific sea-surface temperatures (SSTs), and this connection could be useful for improved forecasting of heat events. As we found in our analysis, large positive anomalies in middle-troposphere geopotential heights are a signature of excessive apparent temperatures in the Southeast and heat events in general in the central and eastern United States [11,12]. These height anomalies have been linked to a pattern of anomalous sea-surface temperatures (SSTs) across the north Pacific Ocean, and the SST pattern provides for skillful prediction of large daily maximum temperatures in the eastern United States up to 50 days in advance [55]. Relatedly, El Niño events tend to decrease the occurrence of extreme heat events in the central United States [56]. As found in this study, summers in the southeastern United States with extremely high ATs and high frequencies of EHDs tend to be preceded by an El Niño event (Fig 8A). For the Southeast as a whole, three summers– 1980, 1998, and 2010 –following an El Niño event had the most EHDs. Anomalously high tropical North Atlantic sea-surface temperatures tend to occur after an El Niño event [57], and this is one possible physical mechanism responsible for extreme summer AT in the Southeast, especially in southeastern Florida, where easterly flow dominates (Fig 4).

**Fig 9:**
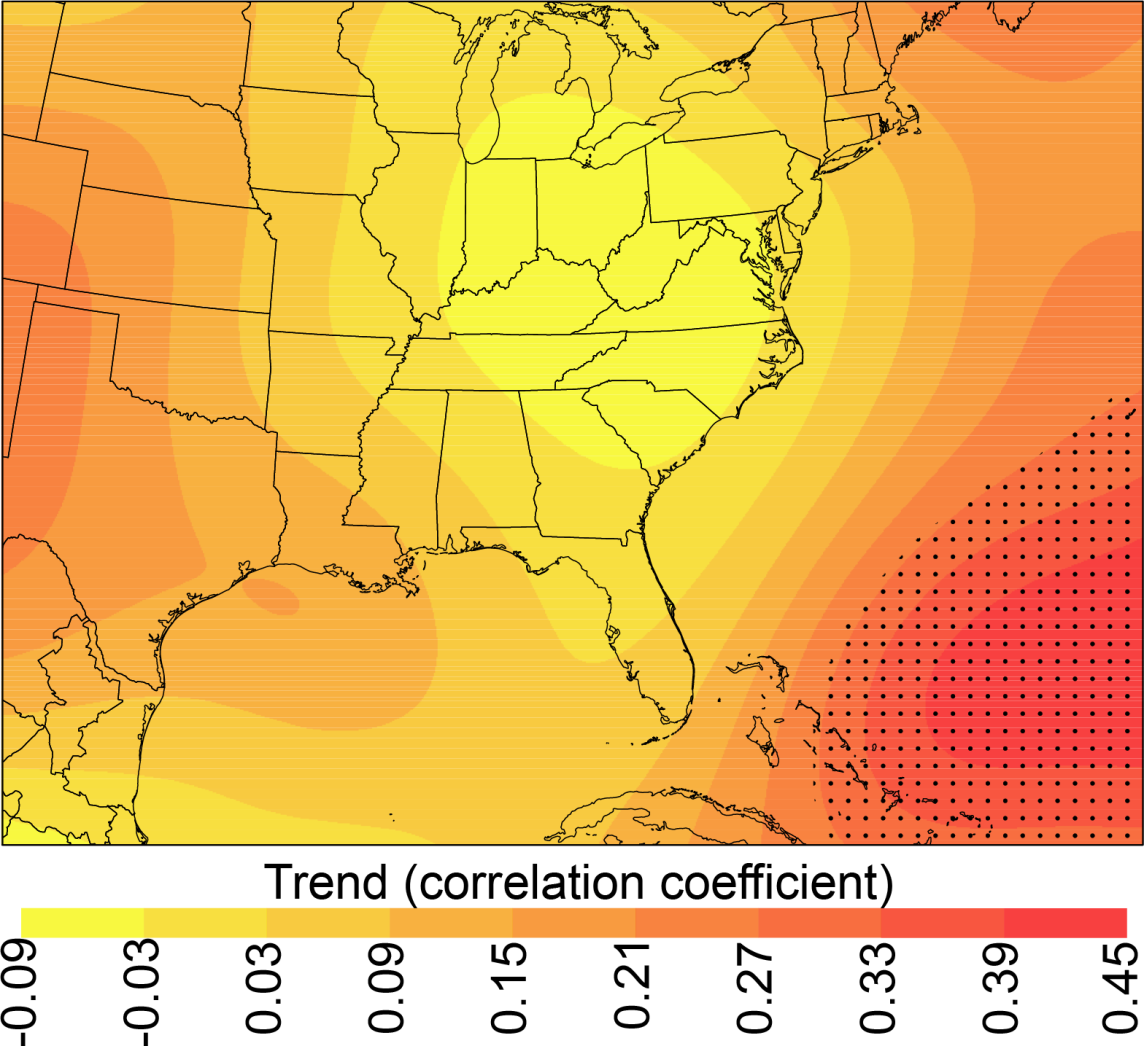
Trend in 500-hPa geopotential heights from 1979–2015. The values are Kendall-Tau correlation coefficients. Significant (α = 0.01; one-tailed) trends are denoted by stippling.

The 1979–2015 trend in AT across the Southeast is considerably smaller than the projected trends based on emissions scenarios. The current trend is just 0.04°C decade^-1^, while the projected trends under the RCP2.6, RCP4.5, and RCP8.5 scenarios are 0.45°C decade^-1^, 0.59°C decade^-1^, and 0.78°C decade^-1^, respectively. The number of EHDs should at least double by 2050, and this is supported by a previous finding of an average six-fold increase in extreme heat events by 2050 for seven southeastern cities [49]. The strong positive correlation between 500-hPa heights and seasonal AT coupled with the lack of a significant increase in warm-season 500-hPa heights over the central and eastern United States during summer (Fig 9) helps to explain the weak increases in summer AT in the Southeast from 1979–2015. GCMs predict a thickening troposphere due to anthropogenic forcings (e.g., increased greenhouse-gas concentrations) and thus increased 500-hPa geopotential heights [58]. The GCM-based findings from this study are supported by results from other studies: based only on temperature, the entire Southeast should have an increase in heat-event temperatures [14,59] and most locales should have a 60-day increase in heat-event frequency by the end of the century [59].

Large urban areas are poised to be the locales most stressed by the increased ATs and associated conditions over the next several decades. Even without correcting for an increasing and aging population, summertime heat-attributable deaths for southeastern cities are projected to double by 2050 [49]. Atlanta and other large urban areas currently have high heat-vulnerability index values resulting from high percentages of the following: (1) population 65 years of age and older, (2) people living alone; (3) race other than white, and (4) people living in poverty [10]. The populations of the cities will increase, with disproportionately large increases in the elderly population; for example, the Atlanta area is projected to grow by 59% by 2030 and thus continue to be the most populous urban area in the Southeast, and a vulnerable population to heat, people 65 years and older, is expected to increase by 200% to approximately 1.2 million people [60].

Concomitant with the increasing ATs should be an increase in the geographical range of climatological conditions conducive to mosquito populations and the spread of emerging and re-emerging vector-borne diseases. *Ae*. *aegypti* populations are projected to spread throughout the Southeast by mid-century, resulting in potential dengue transmission rates equivalent to that of a tropical location (i.e., San Juan, Puerto Rico); peak transmission areas would include all of Florida, the Georgia coastal plain, southern Alabama and Mississippi, and western Mississippi [61]. There is much uncertainty about projected summer rainfall in the Southeast in the coming decades [32]; therefore, it is not known if water availability will be a limiting factor for mosquito populations. Nevertheless, while standing water is necessary for oviposition and larval development, the spread of the dengue virus is ultimately controlled by human contact with mosquitoes [28].

Poor air quality should continue to occur on EHDs within and downwind of urban areas outside of Florida. Summer ozone concentrations have been declining over the past several decades due to decreasing anthropogenic emissions of ozone precursor chemicals [62]. If ozone-precursor emissions return to 1990s or 2000 levels, then summer ozone concentrations will increase by 2050 throughout the Southeast [63,64]. Moreover, it is possible that the 2050 summer climate, with its projected decrease in ventilation, will cause an increase in ozone-pollution episodes, irrespective of changes in precursor emission [65].

The detrimental effects on population health by projected summer warming in the Southeast can be addressed and minimized through thoughtful and innovative structural and behavioral changes as well as dedication to public health preparedness. An extensive implementation of green and cool roofs could actually reduce future temperatures in urban areas even with massive expansions of those urban areas [66]. Ozone concentrations could decrease in a warming climate if anthropogenic ozone-precursor emissions (e.g., nitrogen oxides) continue to decrease through implementation of policies and practices [64]. Increasing standards of living, access to healthcare, and public-health infrastructure would be needed to decrease the effect of heat events and vector-borne diseases on people [67]. For example, improved seasonal forecasting, such as the use of forecasts based on antecedent Pacific SSTs [55], can help southeastern U.S. communities prepare for unhealthy summers both in terms of heat-related morbidity and mortality and in terms of tropical-disease transmission.

## Conclusions

Extremely high summer ATs are common in the southeastern United States and the projected increase in ATs in the coming decades will likely result in increased heat-related health issues. Heat events are caused by shifts towards more anticyclonic circulation patterns, exhibited by increasing middle-troposphere geopotential heights, and that change in circulation is expected to increase over the Southeast in the future. Despite heat acclimatization in the Southeast, positive heat-mortality relationships have been established, and steps are needed to decouple the relationship, especially in the face of projected increases of both AT and heat-vulnerable populations in urban areas. If summer ATs increase without a concomitant large decrease in precipitation, then the geographical spread of mosquito-borne tropical diseases, such as dengue, throughout much of the Southeast appear likely. Mosquito abatement programs are most effective when implemented before there are increases in the mosquito population and the viral agents are introduced.

Three issues related to the interaction of heat and health in the southeastern United States should be addressed. First, the relationship of heat to mortality has been studied in a number of cities in the Southeast, but the relationship of heat to morbidity has received much less attention. Second, the observed trend in AT for the Southeast over the past several decades is much smaller than the projected trends. The predictions are tempered by a disjunction with prior observations, and should be viewed with caution. Third, the presence of unhealthy ground-level ozone concentrations and the transmission of tropical diseases depend on complex processes that are affected by multiple of factors besides temperature. The complexity and detail of climate change requires continued consideration and modeling, but current status still warrants public health action to ameliorate the already established adverse effects of heat damage.

## Supporting information

S1 File Identification numbers, locations, apparent-temperature regions, mean observed (1979–2015) apparent temperature, and mean predicted (2040–2060) apparent temperature for the 71 meteorological stations. (CSV)

S2 File Mean daily apparent temperature for the 71 meteorological stations from 1979–2015. (CSV)

S3 File Mean daily apparent temperature for the eight apparent-temperature region from 1979–2015. (CSV)

S4 File Mean seasonal apparent temperature and frequency of extreme heat days (EHDs) for the eight regions from 1979–2015. (CSV)

S5 File Identification numbers, locations, and mean ozone concentrations for the 71 ozone monitoring stations. (CSV)

S6 File Daily maximum 8-hr average ozone concentrations for the 71 ozone-monitoring stations from 2000–2015. (CSV)

## References

[1] Sherwood SC, Huber M. An adaptability limit to climate change due to heat stress. Proceedings of the National Academy of Sciences. 2010; 107(21):9552–9555. 10.1073/pnas.0913352107 PMC2906879 20439769

[2] Fischer EM, Knutti R. Robust projections of combined humidity and temperature extremes. Nature Climate Change. 2012; 3(2):126–30.

[3] McGeehin MA, Mirabelli M. The potential impacts of climate variability and change on temperature-related morbidity and mortality in the United States. Environmental Health Perspectives. 2001; 109(Supp. 2):185:189. 11359685 10.1289/ehp.109-1240665 PMC1240665

[4] Basu R. High ambient temperature and mortality: a review of epidemiologic studies from 2001 to 2008. Environmental Health. 2009; 8(1):40. 19758453 10.1186/1476-069X-8-40 PMC2759912

[5] Choudhary E, Vaidyanathan A. Heat stress illness hospitalizations—Environmental public health tracking program, 20 states, 2001–2010. Centers for Disease Control and Prevention Morbidity and Mortality Weekly Report Surveillance Summaries. 2014; 63(13):1–10. 25504077

[6] Semenza JC, Rubin CH, Falter KH, Selanikio JD, Flanders WD, Howe HL, et al Heat-related deaths during the July 1995 heat wave in Chicago. New England journal of Medicine. 1996; 335(2):84–90. doi: 10.1056/NEJM199607113350203 8649494 10.1056/NEJM199607113350203

[7] Kalkstein LS, Davis RE. Weather and human mortality: an evaluation of demographic and interregional responses in the United States. Annals of the Association of American Geographers. 1989; 79(1):44–64.

[8] Basu R, Samet JM. Relation between elevated ambient temperature and mortality: a review of the epidemiologic evidence. Epidemiologic Reviews. 2002; 24(2):190–202. 12762092 10.1093/epirev/mxf007

[9] Davis RE, Knappenberger PC, Michaels PJ, Novicoff WM. Changing heat-related mortality in the United States. Environmental Health Perspectives. 2003; 111(14):1712–1718. 14594620 10.1289/ehp.6336 PMC1241712

[10] Maier G, Grundstein A, Jang W, Li C, Naeher LP, Shepherd M. Assessing the performance of a vulnerability index during oppressive heat across Georgia, United States. Weather, Climate, and Society. 2014; 6(2):253–263.

[11] Kunkel KE, Changnon SA, Reinke BC, Arritt RW. The July 1995 heat wave in the Midwest: a climatic perspective and critical weather factors. Bulletin of the American Meteorological Society. 1996; 77(7):1507–1518.

[12] Palecki MA, Changnon SA, Kunkel KE. The nature and impacts of the July 1999 heat wave in the midwestern United States: learning from the lessons of 1995. Bulletin of the American Meteorological Society. 2001; 82(7):1353–1367.

[13] Loikith PC, Broccoli AJ. Characteristics of observed atmospheric circulation patterns associated with temperature extremes over North America. Journal of Climate. 2012; 25(20):7266–7281.

[14] Meehl GA, Tebaldi C. More intense, more frequent, and longer lasting heat waves in the 21st Century. Science. 2004; 305:994–997. doi: 10.1126/science.1098704 15310900 10.1126/science.1098704

[15] Chen F, Konrad CE. A synoptic climatology of summertime heat and humidity in the piedmont region of North Carolina. Journal of applied meteorology and climatology. 2006; 45(5):674–685.

[16] Oke TR. Boundary layer climates London; New York: Routledge; 1992.

[17] Arnfield AJ. Two decades of urban climate research: a review of turbulence, exchanges of energy and water, and the urban heat island. International Journal of Climatology. 2003; 23(1):1–26.

[18] Li D, Bou-Zeid E. Synergistic interactions between urban heat islands and heat waves: the impact in cities is larger than the sum of its parts. Journal of Applied Meteorology and Climatology. 2013; 52(9):2051–2064.

[19] Johnson DP, Wilson JS. The socio-spatial dynamics of extreme urban heat events: the case of heat-related deaths in Philadelphia. Applied Geography. 2009; 29(3):419–434.

[20] Hou P, Wu S. Long-term changes in extreme air pollution meteorology and the implications for air quality. Scientific Reports. 2016; 6:23792 doi: 10.1038/srep23792 27029386 10.1038/srep23792 PMC4815017

[21] Brunekreef B, Holgate ST. Air pollution and health. The Lancet. 360(9341):1233–1242. 10.1016/S0140-6736(02)11274-8 12401268

[22] Comrie AC, Yarnal B. Relationships between synoptic-scale atmospheric circulation and ozone concentrations in metropolitan Pittsburgh, Pennsylvania. Atmospheric Environment Part B Urban Atmosphere. 1992; 26(3):301–312.

[23] Davis JM, Eder BK, Nychka D, Yang Q. Modeling the effects of meteorology on ozone in Houston using cluster analysis and generalized additive models. Atmospheric Environment. 1998; 32(14):2505–2520.

[24] Diem JE, Hursey MA, Morris IR, Murray AC, Rodriguez RA. Upper-level atmospheric circulation patterns and ground-level ozone in the Atlanta metropolitan area. Journal of Applied Meteorology and Climatology. 2010; 49(11):2185–2196.

[25] Cooper OR, Moody JL. Meteorological controls on ozone at an elevated eastern United States regional background monitoring site. Journal of Geophysical Research: Atmospheres. 2000; 105(D5):6855–6869.

[26] Sillman S, Samson F. Impact of temperature on oxidant photochemistry in urban, polluted rural and remote environments. Journal of Geophysical Research-Atmospheres. 1995; 100(D6):11497–11508.

[27] Reid CE, Snowden JM, Kontgis C, Tager IB. The role of ambient ozone in epidemiologic studies of heat-related mortality. Environmental Health Perspectives. 2012; 120(12):1627–1630. doi: 10.1289/ehp.1205251 22899622 10.1289/ehp.1205251 PMC3548272

[28] Eisen L, Monaghan AJ, Lozano-Fuentes S, Steinhoff DF, Hayden MH, Bieringer PE. The impact of temperature on the bionomics of *Aedes* (*Stegomyia*) *aegypti*, with special reference to the cool geographic range margins. Journal of Medical Entomology. 2014; 51(3):496–516. 24897844 10.1603/me13214

[29] Monaghan AJ, Morin CW, Steinhoff DF, Wilhelmi O, Hayden M, Quattrochi DA, et al On the seasonal occurrence and abundance of the Zika virus vector mosquito *Aedes aegypti* in the contiguous United States. PLoS Current Outbreaks. 2016; 10.1371/currents.outbreaks.50dfc7f46798675fc63e7d7da563da76 PMC4807952 27066299

[30] Caminade C, Turner J, Metelmann S, Hesson JC, Blagrove MSC, Solomon T, et al Global risk model for vector-borne transmission of Zika virus reveals the role of El Niño 2015. Proceedings of the National Academy of Sciences. 2017; 114(1):119–124. 10.1073/pnas.1614303114 PMC5224381 27994145

[31] U.S. Census Bureau. Resident population data [Internet]. Washington, DC: U.S. Census Bureau; 2011 [cited 2017 Jan 8]. Available from http://www.census.gov/2010census/data/apportionment-pop-text.php.

[32] Kirtman B, Power SB, Adedoyin JA, Boer GJ, Bojariu R, Camilloni I, et al Near-term climate change: projections and predictability In: Stocker TF, Qin D, Plattner G-K, Tignor M, Allen SK, Boschung J, et al, editors. Climate change 2013: the physical science basis. Contribution of Working Group I to the Fifth Assessment Report of the Intergovernmental Panel on Climate Change. Cambridge, United Kingdom and New York, NY, USA: Cambridge University Press; 2013 pp. 953–1028.

[33] National Weather Service. Meteorological conversions and calculations: heat index calculator [Internet]. Washington, DC: National Weather Service; 2014 5 [cited 2017 Jan 8]. Available from http://www.wpc.ncep.noaa.gov/html/heatindex_equation.shtml

[34] Steadman RG. The assessment of sultriness. Part I: a temperature-humidity index based on human physiology and clothing science. Journal of Applied Meteorology 1979; 18(7):861–873.

[35] Anderson GB, Bell ML, Peng RD. Methods to calculate the heat index as an exposure metric in environmental health research. Environmental Health Perspectives. 2013; 121(10):1111–1119. doi: 10.1289/ehp.1206273 23934704 10.1289/ehp.1206273 PMC3801457

[36] Diem JE, Brown DP. Tropospheric moisture and monsoonal rainfall over the southwestern United States. Journal of Geophysical Research. 2006; 111:D16112.

[37] Mesinger F, DiMego G, Kalnay E, Mitchell K, Shafran PC, Ebisuzaki W, et al North American Regional Reanalysis. Bulletin of the American Meteorological Society. 2006; 87(3):343–360.

[38] Stein AF, Draxler RR, Rolph GD, Stunder BJB, Cohen MD, Ngan F. NOAA’s HYSPLIT atmospheric transport and dispersion modeling system. Bulletin of the American Meteorological Society. 2015; 96(12):2059–2077.

[39] Sheridan SC. The redevelopment of a weather-type classification scheme for North America. International Journal of Climatology. 2002; 22(1):51–68.

[40] Kanamitsu M, Ebisuzaki W, Woollen J, Yang S-K, Hnilo JJ, Fiorino M, et al NCEP–DOE AMIP-II Reanalysis (R-2). Bulletin of the American Meteorological Society. 2002; 83(11):1631–1643.

[41] Helsel DR, Hirsch RM. Statistical methods in water resources In: Hydrologic analysis and interpretation. Washington, DC: U.S. Geological Survey 2002; pp 1–510.

[42] Taylor KE, Stouffer RJ, Meehl GA. An overview of CMIP5 and the experiment design. Bulletin of the American Meteorological Society. 2012; 93(4):485–498.

[43] Abatzoglou JT, Brown TJ. A comparison of statistical downscaling methods suited for wildfire applications. International Journal of Climatology. 2012; 32(5):772–780.

[44] Diem JE. Synoptic-scale controls of summer precipitation in the southeastern United States. Journal of climate. 2006; 19(4):613–621.

[45] Black E, Blackburn M, Harrison G, Hoskins B, Methven J. Factors contributing to the summer 2003 European heatwave. Weather. 2004; 59(8):217–223.

[46] Konrad CE, Fuhrmann CM. Climate of the southeast USA: past, present, and future In: Ingram KT, Dow K, Carter L, Anderson J, editors. Climate of the southeast United States: variability, change, impacts, and vulnerability. Washington, DC: Island Press/Center for Resource Economics; 2013 pp. 8–42.

[47] Florida Department of Environmental Protection. Florida’s ozone and particulate matter air quality trends [Internet]. Tallahassee, FL: Florida Department of Environmental Protection; 2012 12 [cited 2017 March 5]. Available from https://www.dep.state.fl.us/air/air_quality/new_ozone/florida_ozone_pm_trends.pdf

[48] Patz JA, Campbell-Lendrum D, Holloway T, Foley JA. Impact of regional climate change on human health. Nature. 2005; 438:310–317. doi: 10.1038/nature04188 16292302 10.1038/nature04188

[49] Greene S, Kalkstein LS, Mills DM, Samenow J. An examination of climate change on extreme heat events and climate–mortality relationships in large U.S. cities. Weather, Climate, and Society. 2011; 3(4):281–292.

[50] Anderson GB, Bell ML. Heat waves in the United States: mortality risk during heat waves and effect modification by heat wave characteristics in 43 US communities. Environmental Health Perspectives. 2011; 119(2):210–218. doi: 10.1289/ehp.1002313 21084239 10.1289/ehp.1002313 PMC3040608

[51] Curriero FC, Heiner KS, Samet JM, Zeger SL, Strug L, Patz JA. Temperature and mortality in 11 cities of the eastern United States. American Journal of Epidemiology. 2002; 155(1):80–87. 11772788 10.1093/aje/155.1.80

[52] Environmental Protection Agency U.S.. National ambient air quality standards (NAAQS) for ozone [Internet]. Washington DC: U.S. Environmental Protection Agency; 2015 12 [cited 2017 Jan 8]. Available from https://www.epa.gov/ozone-pollution/2015-national-ambient-air-quality-standards-naaqs-ozone.

[53] Aneja VP, Adams AA, Arya SP. An observational based analysis of ozone trends and production for urban areas in North Carolina. Chemosphere-Global Change Science. 2000; 2(2):157–165.

[54] Diem JE. Atmospheric characteristics conducive to high-ozone days in the Atlanta metropolitan area. Atmospheric Environment. 2009; 43(25):3902–3909.

[55] McKinnon KA, Rhines A, Tingley MP, Huybers P. Long-lead predictions of eastern United States hot days from Pacific sea surface temperatures. Nature Geoscience. 2016; 9(5):389–394.

[56] Jia L, Vecchi GA, Yang X, Gudgel RG, Delworth TL, Stern WF, et al The roles of radiative forcing, sea surface temperatures, and atmospheric and land initial conditions in U.S. summer warming episodes. Journal of Climate. 2016; 29(11):4121–4135.

[57] Giannini A, Cane MA, Kushnir Y. Interdecadal changes in the ENSO teleconnection to the Caribbean region and the North Atlantic Oscillation. Journal of Climate. 2001; 14(13):2867–2879.

[58] Santer BD. Behavior of tropopause height and atmospheric temperature in models, reanalyses, and observations: decadal changes. Journal of Geophysical Research. 2003; 301: 479–483.

[59] Kunkel KE, Liang X-Z, Zhu J. Regional climate model projections and uncertainties of U.S. summer heat waves. Journal of Climate. 2010; 23(16):4447–4458.

[60] Hildner KF, Nichols A, Martin S. Methodology and assumptions for the Mapping America’s Futures Project. Mapping America’s Futures, Brief 5. 2015 Available from http://www.urban.org/sites/default/files/publication/33931/2000069-Methodology-and-Assumptions-for-Mapping-Americas-Futures-Project.pdf

[61] Butterworth MK, Morin CW, Comrie AC. An analysis of the potential impact of climate change on dengue transmission in the southeastern United States. Environmental Health Perspectives. 2016. 10.1289/EHP218 PMC5381975 27713106

[62] Zhang Y, Wang Y. Climate-driven ground-level ozone extreme in the fall over the Southeast United States. Proceedings of the National Academy of Sciences. 2016; 113(36):10025–10030. 10.1073/pnas.1602563113 PMC5018760 27551089

[63] Bell ML, Goldberg R, Hogrefe C, Kinney PL, Knowlton K, Lynn B, et al Climate change, ambient ozone, and health in 50 US cities. Climatic Change. 2007; 82(1–2):61–76.

[64] Pfister GG, Walters S, Lamarque J-F, Fast J, Barth MC, Wong J, et al Projections of future summertime ozone over the U.S. Journal of Geophysical Research: Atmospheres. 2014; 119:5559–5582.

[65] Wu S, Mickley LJ, Leibensperger EM, Jacob DJ, Rind D, Streets DG. Effects of 2000–2050 global change on ozone air quality in the United States. Journal of Geophysical Research. 2008; 113:D06302.

[66] Georgescu M, Morefield PE, Bierwagen BG, Weaver CP. Urban adaptation can roll back warming of emerging megapolitan regions. Proceedings of the National Academy of Sciences. 2014; 111(8):2909–2914. 10.1073/pnas.1322280111 PMC3939866 24516126

[67] Gubler DJ, Reiter P, Ebi KL, Yap W, Nasci R, Patz JA. Climate variability and change in the United States: potential impacts on vector- and rodent-borne diseases. Environmental Health Perspectives. 2001; 109:223–233. 11359689 10.1289/ehp.109-1240669 PMC1240669

